# Optically locked low-noise photonic microwave oscillator

**DOI:** 10.1093/nsr/nwag135

**Published:** 2026-03-16

**Authors:** Zexing Zhao, Bin Li, Kunpeng Jia, Chenye Qin, Xiaofan Zhang, Jingru Ji, Hua-Ying Liu, Xiao-Hui Tian, Zhong Yan, Xiaoshun Jiang, Xinlun Cai, Biaobing Jin, Zhenlin Wang, Wei Liang, Shi-ning Zhu, Zhenda Xie

**Affiliations:** National Laboratory of Solid State Microstructures, School of Electronic Science and Engineering, College of Engineering and Applied Sciences, School of Physics, Research Institute of Superconductor Electronics (RISE) & Key Laboratory of Optoelectronic Devices and Systems with Extreme Performances of MOE, Key Laboratory of Intelligent Optical Sensing and Manipulation, Ministry of Education, and Collaborative Innovation Center of Advanced Microstructures, Nanjing University, Nanjing 210093, China; National Laboratory of Solid State Microstructures, School of Electronic Science and Engineering, College of Engineering and Applied Sciences, School of Physics, Research Institute of Superconductor Electronics (RISE) & Key Laboratory of Optoelectronic Devices and Systems with Extreme Performances of MOE, Key Laboratory of Intelligent Optical Sensing and Manipulation, Ministry of Education, and Collaborative Innovation Center of Advanced Microstructures, Nanjing University, Nanjing 210093, China; National Laboratory of Solid State Microstructures, School of Electronic Science and Engineering, College of Engineering and Applied Sciences, School of Physics, Research Institute of Superconductor Electronics (RISE) & Key Laboratory of Optoelectronic Devices and Systems with Extreme Performances of MOE, Key Laboratory of Intelligent Optical Sensing and Manipulation, Ministry of Education, and Collaborative Innovation Center of Advanced Microstructures, Nanjing University, Nanjing 210093, China; National Laboratory of Solid State Microstructures, School of Electronic Science and Engineering, College of Engineering and Applied Sciences, School of Physics, Research Institute of Superconductor Electronics (RISE) & Key Laboratory of Optoelectronic Devices and Systems with Extreme Performances of MOE, Key Laboratory of Intelligent Optical Sensing and Manipulation, Ministry of Education, and Collaborative Innovation Center of Advanced Microstructures, Nanjing University, Nanjing 210093, China; National Laboratory of Solid State Microstructures, School of Electronic Science and Engineering, College of Engineering and Applied Sciences, School of Physics, Research Institute of Superconductor Electronics (RISE) & Key Laboratory of Optoelectronic Devices and Systems with Extreme Performances of MOE, Key Laboratory of Intelligent Optical Sensing and Manipulation, Ministry of Education, and Collaborative Innovation Center of Advanced Microstructures, Nanjing University, Nanjing 210093, China; Key Laboratory of Semiconductor Display Materials and Chips, Suzhou Institute of Nano-Tech and Nano-Bionics, Chinese Academy of Sciences, Suzhou 215213, China; National Laboratory of Solid State Microstructures, School of Electronic Science and Engineering, College of Engineering and Applied Sciences, School of Physics, Research Institute of Superconductor Electronics (RISE) & Key Laboratory of Optoelectronic Devices and Systems with Extreme Performances of MOE, Key Laboratory of Intelligent Optical Sensing and Manipulation, Ministry of Education, and Collaborative Innovation Center of Advanced Microstructures, Nanjing University, Nanjing 210093, China; National Laboratory of Solid State Microstructures, School of Electronic Science and Engineering, College of Engineering and Applied Sciences, School of Physics, Research Institute of Superconductor Electronics (RISE) & Key Laboratory of Optoelectronic Devices and Systems with Extreme Performances of MOE, Key Laboratory of Intelligent Optical Sensing and Manipulation, Ministry of Education, and Collaborative Innovation Center of Advanced Microstructures, Nanjing University, Nanjing 210093, China; Nanzhi Institute of Advanced Optoelectronic Integration, Nanjing 211800, China; National Laboratory of Solid State Microstructures, School of Electronic Science and Engineering, College of Engineering and Applied Sciences, School of Physics, Research Institute of Superconductor Electronics (RISE) & Key Laboratory of Optoelectronic Devices and Systems with Extreme Performances of MOE, Key Laboratory of Intelligent Optical Sensing and Manipulation, Ministry of Education, and Collaborative Innovation Center of Advanced Microstructures, Nanjing University, Nanjing 210093, China; State Key Laboratory of Optoelectronic Materials and Technologies, School of Electronics and Information Technology, Sun Yat-sen University, Guangzhou 510275, China; National Laboratory of Solid State Microstructures, School of Electronic Science and Engineering, College of Engineering and Applied Sciences, School of Physics, Research Institute of Superconductor Electronics (RISE) & Key Laboratory of Optoelectronic Devices and Systems with Extreme Performances of MOE, Key Laboratory of Intelligent Optical Sensing and Manipulation, Ministry of Education, and Collaborative Innovation Center of Advanced Microstructures, Nanjing University, Nanjing 210093, China; Purple Mountain Laboratories, Nanjing 211111, China; National Laboratory of Solid State Microstructures, School of Electronic Science and Engineering, College of Engineering and Applied Sciences, School of Physics, Research Institute of Superconductor Electronics (RISE) & Key Laboratory of Optoelectronic Devices and Systems with Extreme Performances of MOE, Key Laboratory of Intelligent Optical Sensing and Manipulation, Ministry of Education, and Collaborative Innovation Center of Advanced Microstructures, Nanjing University, Nanjing 210093, China; Key Laboratory of Semiconductor Display Materials and Chips, Suzhou Institute of Nano-Tech and Nano-Bionics, Chinese Academy of Sciences, Suzhou 215213, China; National Laboratory of Solid State Microstructures, School of Electronic Science and Engineering, College of Engineering and Applied Sciences, School of Physics, Research Institute of Superconductor Electronics (RISE) & Key Laboratory of Optoelectronic Devices and Systems with Extreme Performances of MOE, Key Laboratory of Intelligent Optical Sensing and Manipulation, Ministry of Education, and Collaborative Innovation Center of Advanced Microstructures, Nanjing University, Nanjing 210093, China; National Laboratory of Solid State Microstructures, School of Electronic Science and Engineering, College of Engineering and Applied Sciences, School of Physics, Research Institute of Superconductor Electronics (RISE) & Key Laboratory of Optoelectronic Devices and Systems with Extreme Performances of MOE, Key Laboratory of Intelligent Optical Sensing and Manipulation, Ministry of Education, and Collaborative Innovation Center of Advanced Microstructures, Nanjing University, Nanjing 210093, China

**Keywords:** micro Fabry-Pérot cavity, thin-film lithium niobate electro-optic comb, optically locked reference lasers, optical frequency division, low-noise photonic microwave generation

## Abstract

The next generation of sensing and communication applications rely on high-frequency microwave generation with low noise. Microwave photonic technology is highly promising; however, its practical applications have so far been limited by the complexity of existing system architectures. Here, we demonstrate an optically locked low-noise photonic microwave oscillator, in which all the optical components are packaged within a small module of 166 mL; low noise microwave generation is achieved at 10.4 GHz with single-sideband phase noise of −54 dBc/Hz at 10 Hz, −141 dBc/Hz at 10 kHz, and −162 dBc/Hz at 10 MHz offset. The above performance arises from a dual-laser self-injection-locking scheme to a single Fabry–Pérot cavity with high Q exceeding 10^8^, with >20 dB common-mode noise suppression. The low-noise nature of this reference is coherently transferred to the X-band through a high-performance thin-film lithium niobate (TFLN) electro-optic comb chip, thereby overcoming long-standing barriers in photonic microwave integration to enable truly field-deployable low-noise microwave generation.

## Teaser

Integrated photonic microwave oscillator matches the noise performance of big optical-frequency-division systems (-141 dBc/Hz, 10 kHz offset at 10 GHz). Optically-locked dual-lasers and TFLN circuit package is within 166 mL.

## INTRODUCTION

High-performance microwave generation defines capabilities of the electronic communication, radar, and sensing systems. The increasing demand for high communication speed and sensing precision push microwaves to high operating frequency and low phase noise simultaneously. These stringent requirements pose fundamental challenges for conventional electronic technologies, whose noise performance typically degrades as the operating frequency increases. Microwave photonic techniques leverage the inherently high frequencies of optical oscillators to break this compromise [1–3], transferring the high frequency and low phase noise stability of optical reference into the microwave domain with high fidelity [4–7], albeit at the cost of much greater system complexity in locking compared to electronic methods. Acting as the ultimate ‘phase anchor’ for photonic microwave oscillators, conventional optical references typically rely on large-scale ultra-stable cavities [8]. Their vacuum housings and active thermal isolation systems lead to substantial system size and power consumption, which hinders miniaturization and flexible deployment. This has become a critical bottleneck limiting further practical applications.

To translate high-performance photonic microwave oscillators into practical systems and fully leverage the advantages of photonic integration, optical references need to be implemented in more compact architectures [9–12]. Recently, ultra-high-Q miniature Fabry–Pérot (mini-FP) reference cavities [13–15] offer an absolute optical frequency reference with much lower thermorefractive noise (TRN) and longer photon lifetimes than conventional dielectric resonators [16–19]. However, realizing optical reference–based photonic microwave oscillators (OR-PMOs) with such ultra-stable FP reference cavity faces several challenges: active locking requires real-time electrical feedback, which leads to bulky and power-hungry architectures, and the FP reference cavity is inherently difficult to integrate into compact, deployable systems. Developing miniaturized optical references with intrinsic frequency stability is expected to pave the way for integrated and deployable low-noise photonic microwave oscillators.

In this work, we demonstrate a compact low-noise photonic microwave oscillator based on an optically locked micro optical reference. Two distributed-feedback (DFB) lasers are simultaneously self-injection locked to a single ultra-high-Q micro Fabry–Pérot (μFP) optical reference cavity with a volume of only 1 mL. This fully optical locking scheme generates dual ultra-narrow-linewidth lasers, with each laser exhibiting a fundamental linewidth of only a few millihertz. By self-injection-locking both lasers to the same μFP cavity, phase-noise fluctuations induced by cavity vibrations and thermal noise are largely suppressed. As a result, >20 dB of common-mode noise suppression (CMNS) is achieved. An integrated high-performance thin-film lithium niobate (TFLN) cascaded modulator is used to generate dual electro-optic (EO) frequency combs, serving as the frequency-dividing element in the electro-optical-frequency-division (e-OFD) system. The e-OFD scheme transfers the stabilized optical reference to the X-band, yielding a 10.4 GHz carrier with single-sideband (SSB) phase noise of −54 dBc/Hz at 10 Hz, −141 dBc/Hz at 10 kHz, and −162 dBc/Hz at 10 MHz offset frequency. The oscillator further supports continuous frequency tuning over 350 kHz without mode hopping, highlighting its potential as a compact and deployable platform for high-performance photonic microwave systems.

## RESULTS

### Compact low-noise photonic microwave oscillator system

The architecture of the compact low-noise photonic microwave oscillator system based on e-OFD (Fig. 1) comprises a frequency-stabilized micro optical reference, an on-chip frequency divider, and a microwave signal generator. The e-OFD scheme employs the frequency divider to phase-lock a tunable electronic oscillator to the optical reference, thereby generating low-noise microwave signals. A key advantage of e-OFD is that all signal processing takes place within the bandwidth of standard electronic equipment, enabling precise tuning to achieve the desired microwave frequency.

**Figure 1.**
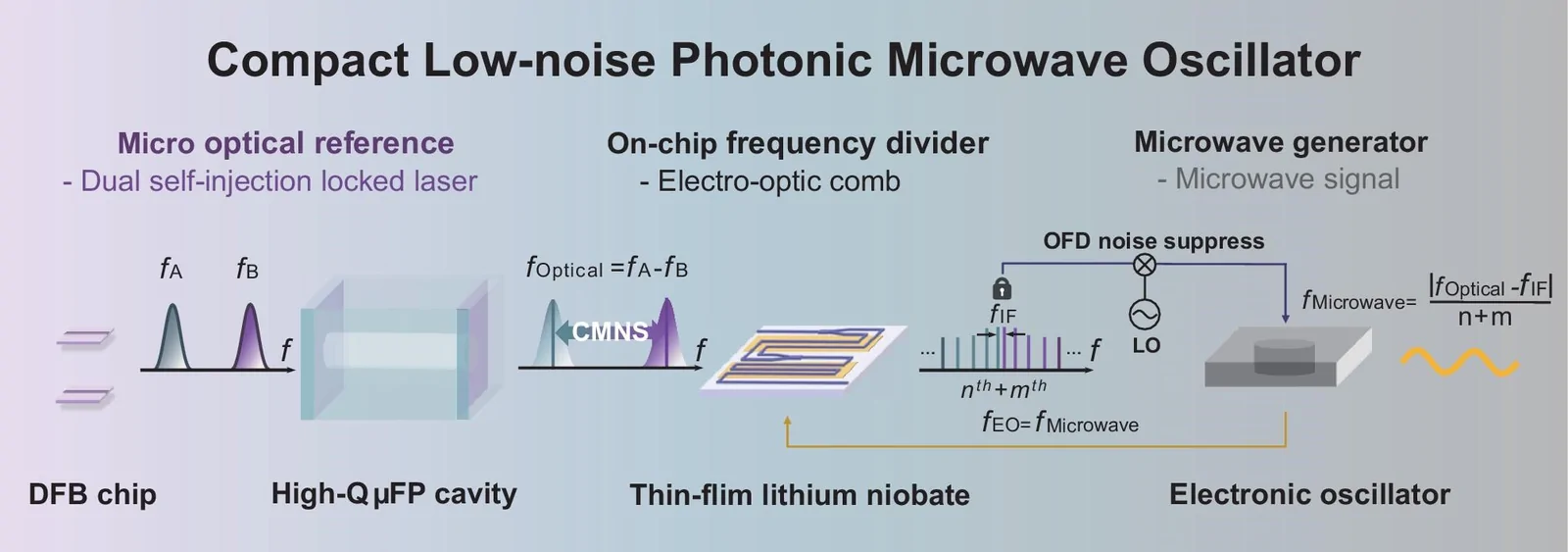
Concept of compact low-noise photonic microwave oscillator based on high-Q μ-Fabry–Pérot (μFP) reference cavity. The photonic microwave oscillator system is mainly composed of three parts. (i) Micro optical reference. A miniature micro-optics system was constructed to produce a dual self-injection locked laser with high common-mode noise suppression (CMNS) which serves as a frequency-stabilized micro optical reference. (ii) On-chip frequency divider. High-performance thin-film lithium niobate cascade modulator generates electro-optic combs. New intermediate frequency signals are produced at the two nearest dual EO combs with amplified phase noise information. (iii) Microwave generator. The tunable electronic oscillator generates microwave signals used to drive the modulator. The error signal generated by the intermediate frequency signal and the local oscillator (LO) signal is fed back to the oscillator through servo control to form a microwave oscillation loop, generating a low-noise microwave signal.

At the core of this system is a compact dual self-injection-locked (dSIL) laser that provides the high-performance optical frequency reference. The high-Q external-cavity-based self-injection locking technique enables optical locking between the laser and the reference cavity [20,21] and achieves strong linewidth compression [22–24] without the need for additional electrical feedback loops [25]. In the dSIL configuration, two DFB pump lasers are coupled to the μFP cavity through a micro-optics system. Owing to the ultra-high-Q μFP cavity’s frequency selectivity, the fundamental linewidths of the lasers will be significantly compressed by several orders of magnitude, which is crucial for ensuring frequency stability of the optical reference.

The high-performance TFLN modulator generates dual electro-optic (EO) combs that act as frequency dividers. These combs coherently subdivide the stabilized optical reference into equally spaced lines, enabling optical-to-microwave frequency division within the e-OFD system. In recent years, the TFLN platform has demonstrated significant research results in χ^(3)^- [26–28] and χ^(2)^-based devices [29,30]. The most distinctive capability of TFLN is the high Pockels coefficient, which enables high-performance EO modulation that is critical for bridging microwave and optical domains. TFLN thus has facilitated electro-optic integrated devices including low half-wave voltage (V_π_) modulators [31], EO frequency combs [32,33], and high-speed tunable lasers [34,35], driving progress in optical computing [36], precise detection [37,38], and microwave photonics [39,40]. As the premier electrical-to-optical interface platform, high-performance TFLN is essential for realizing integrated e-OFD technology.

Subsequently, the dSIL lasers are coupled into a high-performance TFLN modulator driven by an external free-running electronic oscillator, generating dual EO combs with identical repetition frequency. The two nearest comb lines in the center of the EO combs produce an intermediate frequency signal ${f}_{IF} = ({f}_B - m{f}_{EO}) - ({f}_A + n{f}_{EO})$. The intermediate frequency signal accumulates phase noise jitter scaled by a factor of (*n* + *m*) relative to the oscillator [7]. By optimizing microwave parameters, the intermediate frequency signal can be mixed with a stable low-frequency local oscillator. The down-converted error signal then drives a servo feedback loop that divides the original optical reference into the RF domain through an (*n* + *m*)-fold frequency division process. Correspondingly, the original phase noise is suppressed by a factor of ${{( n + m)}^2} $, enabling the generation of low-noise microwave signals.

### Micro-photonics device design

The air-gap high-Q μFP cavity-based dSIL lasers are essential for obtaining a frequency-stabilized optical reference. Critical to dSIL performance, the μFP cavity is fabricated from ultra-low thermal-expansion glass (Zerodur), effectively minimizing thermally induced fluctuations of cavity length. Compared with mini-FP cavities, the μFP achieves comparable Q factors and low thermorefractive noise in a significantly smaller footprint, making its physical scale highly compatible with photonic-chip integration [15]. As depicted in Fig. 2a (with the corresponding optical photograph in Fig. 2d), this air-gap high-Q μFP cavity structure inherently suppresses the TRN-induced frequency drift. Fabrication commenced with ultra-smooth polishing and high-reflection coating of two square plates, which are then assembled to form a hollow FP cavity. The cavity dimensions are 9 mm × 9 mm × 13 mm, corresponding to a volume of ∼1 mL. The free spectral range (FSR) near 1550 nm is 15 GHz and the Q factor is measured to be >10^8^ (Fig. 2e).

**Figure 2.**
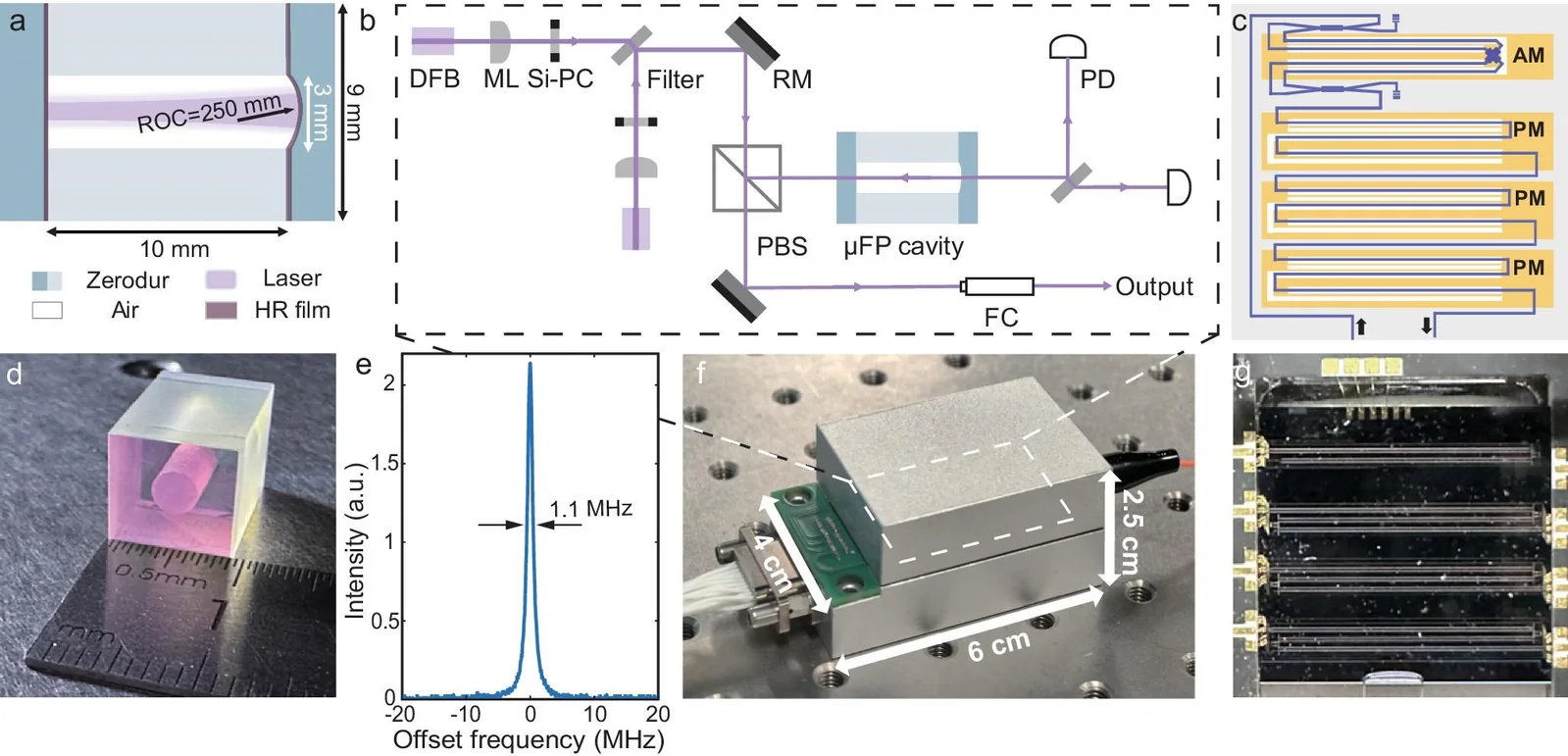
Device architecture and micro-photonics platform. (a) Schematic of the high-Q μFP cavity. ROC, radius of curvature. (b) Schematic of the micro-optics system for generating dSIL lasers based on μFP cavity. ML, micro-lens. Si-PC, silicon-based phase controller. RM, reflect mirror. PBS, polarization beam splitter. PD, photodetector. FC, fiber collimator. (c) TFLN cascade modulator architecture. (d) Optical photograph of the μFP cavity. (e) Q-factor characterization at 1550 nm. (f) Optical photograph of the dSIL lasers’ packaging module; white dashed area: micro-optics system in (b). (g) Optical photograph of the integrated TFLN modulator.

We achieve the optically locked, frequency-stabilized micro optical reference by building a compact dSIL system (Fig. 2b). Two commercial DFB diode lasers centered at 1548.3 nm and 1553.3 nm respectively are employed, with output power of 80 mW and fundamental linewidth of ∼100 kHz. Each DFB laser is first collimated by a microlens and then directed onto a filter after passing through a silicon-based phase controller. The filter is designed to have a high transmission for one laser and a high reflection for the other, enabling both lasers to be coupled into the μFP cavity through the same optical path while avoiding crosstalk during the SIL process. After reflection and beam splitting, the DFB lasers are coupled into the μFP cavity. The transmitted light passes through the filter and is spatially aligned with two photodetector chips. These photodetectors independently monitor the SIL states of the two lasers. Meanwhile, the reflected light returns to the respective DFB chips along the original optical path. Through this process, dual ultra-narrow-linewidth self-injection-locked lasers are generated and delivered to an optical fiber coupler, providing a total output power of 3 mW. The linewidth narrowing coefficient η is given by the following formula [41]:


(1) \begin{eqnarray*}
{\mathrm{\eta }} \approx \frac{{Q_{DFB}^2}}{{Q_{FP}^2}}\frac{1}{{16{\mathrm{\Gamma }}_{FP}^2\left( {1 + \alpha _g^2} \right)}},
\end{eqnarray*}

where Q factor of the DFB chip ${Q}_{DFB}$ is 4∼6 × 10^3^, Q factor of the μFP, ${Q}_{FP}$ is 2 × 10^8^, amplitude-phase coupling coefficient ${\alpha }_g$ is 2.5, and the reflection coefficient ${{\mathrm{\Gamma }}}_{FP}$ in our system is 1∼3 × 10^−2^. These parameters are influenced by the manufacturing tolerances of commercial DFB chips, the optical-path coupling efficiency, and variations among the micro-optics components. As a result, the linewidth narrowing factor η falls with the range of 10^−7^ to 10^−8^.

We implement an integrated circuit for convenient connection to external leads with the modular packaging (Fig. 2f). The overall volume is ∼60 mL with a total weight of 113 g. Notably, the entire micro-optical subsystem occupies only 18 mm × 34 mm × 9 mm (white dashed frame in Fig. 2f), while the remaining portion of the module consists of a circuit board for external connections and a protective metal enclosure. The system is pumped by external electric circuits to control the DFB lasers, silicon phase shifters, and TEC temperature, thereby enabling both the generation and state control of the dSIL lasers. The SIL status can be monitored through the embedded PD electrical signal output, thereby ensuring robust self-injection locking. This compact, modular packaging enables the deployment of stable optical-reference sources for distributed applications.

For EO combs generation, we employ a TFLN modulator (as shown as Fig. 2c, g) featuring a cascaded Mach-Zehnder modulator (MZM) and three phase modulators (PMs) in series. This architecture significantly reduces RF power requirements per phase modulator while supporting broadband EO comb generation under low-frequency driving conditions. Owing to an efficient optically and electrically folded design (details in Ref. [42]), the TFLN device achieves a low half-wave voltage (≈1.7 V @ low-frequency for MZM, ≈2 V for PMs). Both optical and RF ports are also packaged within a miniaturized module of ∼106 mL for system integration.

### Optically locked frequency-stabilized micro optical reference

We experimentally validate the performance of the packaged ultra-narrow-linewidth dSIL laser module as a turnkey, high-performance frequency-stabilized micro optical reference (Fig. 3). The characterization through the testing setup is shown in Fig. 3a. Crucially, the dSIL processes within this linear cavity can basically be regarded as independent of each other [43] and are inherently polarization insensitive. This enables independent adjustment of each laser’s locking state without cross-interference, thereby significantly enhancing control flexibility.

**Figure 3.**
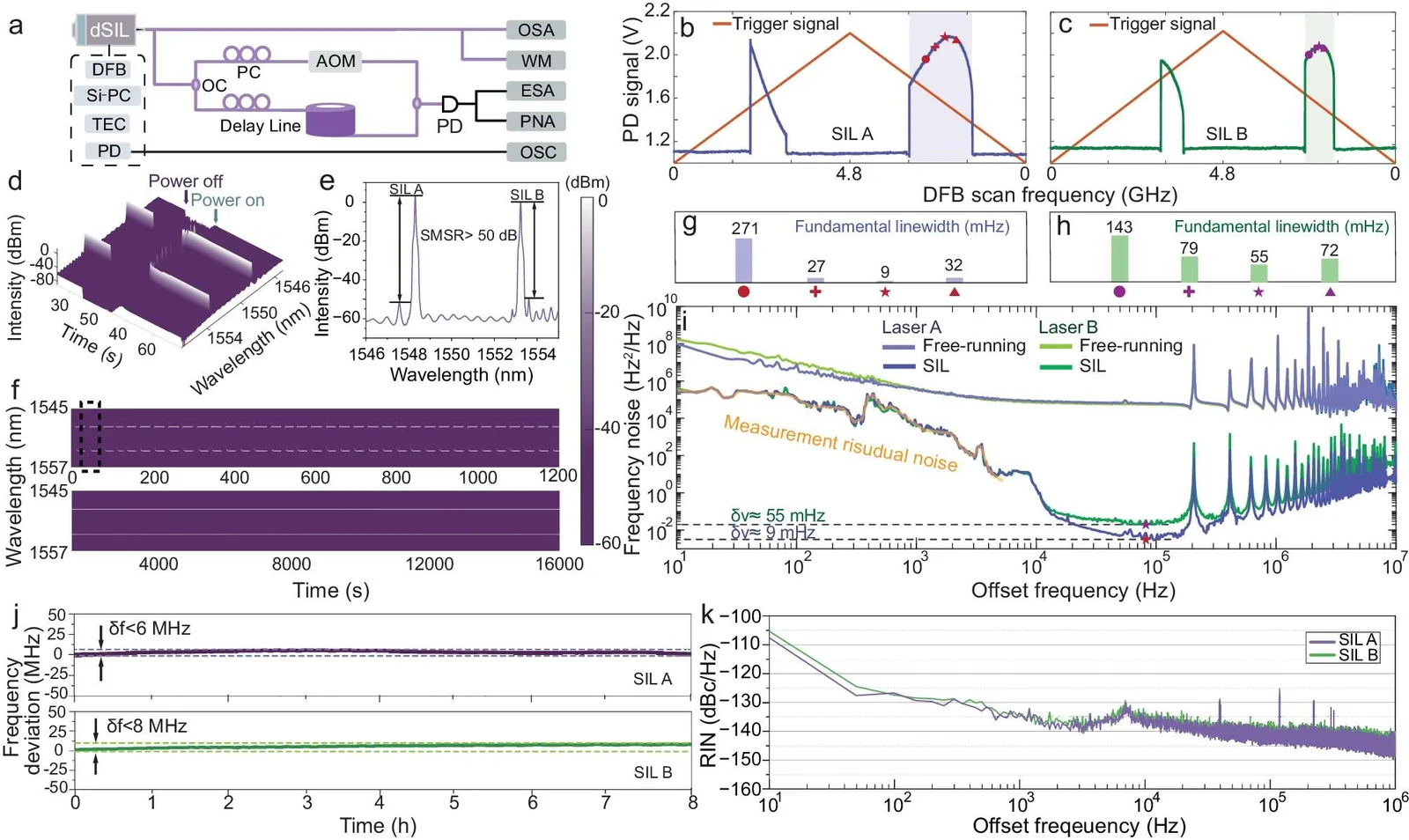
dSIL laser characterization and noise performance. (a) The dSIL laser test setup. TEC, thermo-electric-cooler. OC, optical coupler. PC, polarization controller. AOM, acousto-optic modulator. OSA, optical spectrum analyzer. WM, wavelength meter. ESA, electrical spectrum analyzer. PNA, phase noise analyzer. OSC, oscilloscope. (b and c) The detected PDs’ monitor signals of dSIL lasers’ module when scanning the driven current. (d) The spectral evolution of the turnkey dSIL lasers (black dashed box in (f)). (e) Optical spectra of dSIL lasers. (f) The spectrum evolution of dSIL lasers in consecutive turnkey operation (upper) and in long-term free-running state (lower). (g and h) Fundamental linewidth variation (locations marked in (b) and (c)). (i) The frequency noise characterization of the lasers in free-running and SIL states (resonance center). Dashed horizontal line indicates noise floor for fundamental linewidth calculation. (j) Long-term frequency drift measured by WM. (k) The measured RIN of dSIL lasers.

By applying a triangular-wave modulation to the driven currents of DFB lasers and monitoring the real-time electrical outputs from the embedded photodetectors, we observe clear power transitions associated with the locking and unlocking events of SIL A and SIL B (Fig. 3b and c). The measured self-injection locking bandwidths are ∼1.77 GHz for SIL A and 0.82 GHz for SIL B. We evaluate the turnkey operation of the dSIL laser module. The optical-reference system reliably initiates and terminates simultaneous self-injection locking of both lasers at 1548.3 nm and 1553.3 nm (Fig. 3d and f upper panel). The dSIL lasers exhibit a side-mode suppression ratio (SMSR) exceeding 50 dB (Fig. 3e) and achieves long-term self-stable maintenance (Fig. 3f lower panel).

We characterize the linewidths of the dSIL lasers using a delay-based heterodyne self-frequency system (see details in Methods) [44,45]. Within the detuning range of ∼300 MHz, the fundamental linewidths of the dSIL lasers remain stable, demonstrating exceptional robustness for optical reference applications (Fig. 3g, h). The results of the frequency noise of both lasers in the free-running and SIL states are shown in Fig. 3i. The fundamental linewidths of the SIL laser A and SIL laser B are 9 mHz and 55 mHz, respectively, which are derived from the base of the frequency noise curves [44]. We further use a wavelength meter (Highfinesse WS-U) to assess the long-term frequency stability of the SIL lasers. Thanks to the stable frequency optical locking enabled by the air-gap high-Q microcavity and the mechanical rigidity of the packaged module, the central frequencies of the both SIL lasers exhibit a drift of only a few megahertz over an 8-hour duration. The relatively intensity noise (RIN) of the dSIL lasers (by using Rohde & Schwarz FSV30) are demonstrated in Fig. 3k. Both RIN curves fall below −135 dB/Hz beyond 10 kHz offset, confirming the excellent power stability of dSIL lasers as optical reference.

Notably, by simultaneously locking two lasers to a common μFP in a compact configuration, the scheme naturally rejects common-mode noise arising from cavity vibrations and thermal fluctuations in the dSIL laser system. As a result, the heterodyne beat note between the two SIL lasers directly reveals the effectiveness of CMNS. The phase noise of the dual SIL lasers’ beat note (as shown in Fig. 4c) is measured via a two-wavelength delayed self-heterodyne interferometer (TWDI, measured details in Methods). Compared with the individual SIL laser phase noise (grey curves), the CMNS of the dual SIL lasers’ beat note reached ∼20 dB (pink curve).

**Figure 4.**
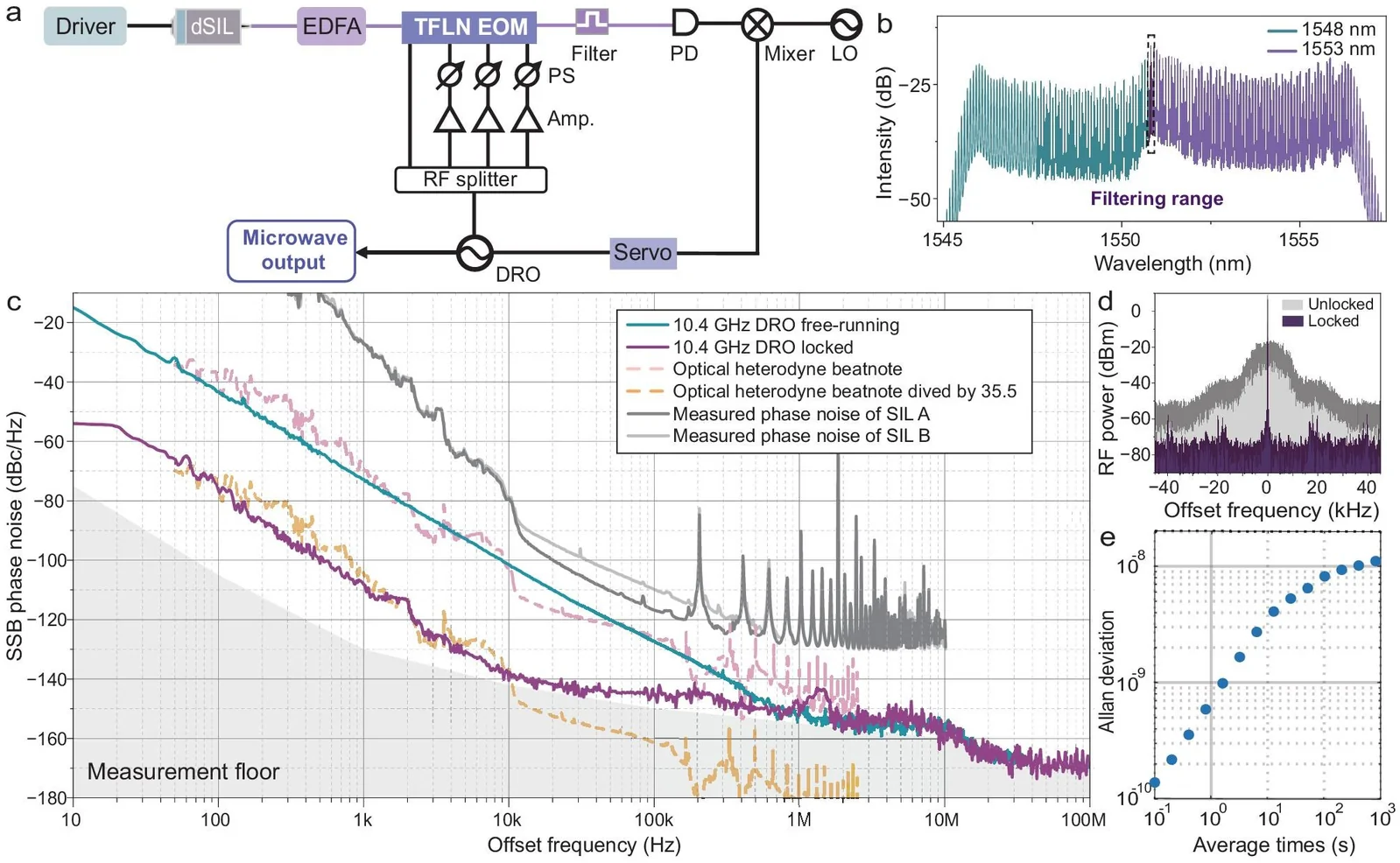
Compact electro-optic frequency division and microwave synthesis. (a) Schematic of compact e-OFD system. EDFA, erbium-doped fiber amplifier. LO, local oscillator. PS, phase shifter. DRO, dielectric resonator oscillator. (b) Dual EO combs spectrum. The dotted line area indicates comb line filtered for photodetection to generate an intermediate frequency signal. (c) SSB phase noise of the 10.4 GHz microwave signal in both free-running and locked states. The phase noise of the optical reference is obtained using optical heterodyne detection, and the corresponding phase noise after e-OFD is calculated. The phase noise of the original dual free-running lasers was plotted for comparison. (d) The electrical spectra of intermediate frequency signals in both locked and unlocked states. (e) Allan deviation of the locked 10.4 GHz DRO.

### Low-noise tunable microwave signal generation

Figure 4a illustrates the microwave-optical oscillation circuit of the e-OFD photonic microwave generation system. The dSIL laser module first generates dSIL lasers at 1548.3 nm and 1555.3 nm, corresponding to an optical bandwidth of 625 GHz for the OFD. The dSIL lasers are then optically amplified and coupled into the packaged, integrated TFLN cascade modulator. An external dielectric resonator oscillator (DRO) RF source provides a 10.4 GHz microwave signal. The microwave signal is transmitted into the TFLN cascade modulator through the RF link (with a total loaded RF power of ∼35 dBm), generating dual EO combs (Fig. 4b). The theoretical noise-suppression level achieved through frequency division, given by log_10_($\frac{{625\ GHz}}{{10.4\ GHz}}$)^2^ = 35.5 dB, is expected. We use an optical filter to select adjacent comb lines from the dual EO combs, which are detected by a photodetector to produce an intermediate frequency (${{\mathrm{f}}}_{IF} \approx 100{\mathrm{\ MHz}}$). This signal is then mixed with a 100 MHz local oscillator (LO) to generate an error signal, which is fed into the servo controller and subsequently applied as feedback to the DRO. Once the ${{\mathrm{f}}}_{IF}$ signal is locked, the power spectral density of the microwave phase noise is suppressed by the square of the frequency-division factor over the frequency range within the servo’s control bandwidth.

We use a phase noise analyzer to characterize the phase noise of the output microwave signals in both the free-running and locked states (Fig. 4c). The SSB phase noise of the locking 10.4 GHz signal from the compact low-noise photonic microwave oscillation system achieves −54 dBc/Hz at 10 Hz offset, −77 dBc/Hz at 100 Hz, −108 dBc/Hz at 1 kHz, −141 dBc/Hz at 10 kHz, −146 dBc/Hz at 100 kHz, −152 dBc/Hz at 1 MHz, and −162 dBc/Hz at 10 MHz. Dividing the beat note phase noise by the frequency division factor of 35.5 dB yields excellent agreement with the measured microwave phase noise, with minor deviations above 10 kHz attributed to PNA measurement limitations and servo-feedback bandwidth constraints. Nevertheless, the optically locked frequency-stabilized optical reference enables exceptional phase noise performance beyond 10 kHz offset. The fractional frequency stability of the output microwave signal is presented in Fig. 4e.

We further investigate the frequency tunability of the low-noise photonic microwave oscillator (Fig. 5). The output microwave frequency is equal to $\frac{{{f}_B - {f}_A - {f}_{IF}}}{{m + n}}$, where ${f}_{IF}$ is locked to the local oscillator reference. By adjusting the local oscillator frequency, the output microwave frequency can be precisely tuned. A triangular-wave modulation with a period of 100 Hz is applied to the local oscillator, producing a 21 MHz frequency deviation in the reference. The locking system tracks this modulation, resulting in a corresponding microwave frequency deviation of $\frac{{21{\mathrm{\ MHz}}}}{{60}} = 350{\mathrm{\ \ kHz}}$ in the low-noise microwave output. Notably, the tuning bandwidth is only limited by the tuning range of the local oscillator.

**Figure 5.**
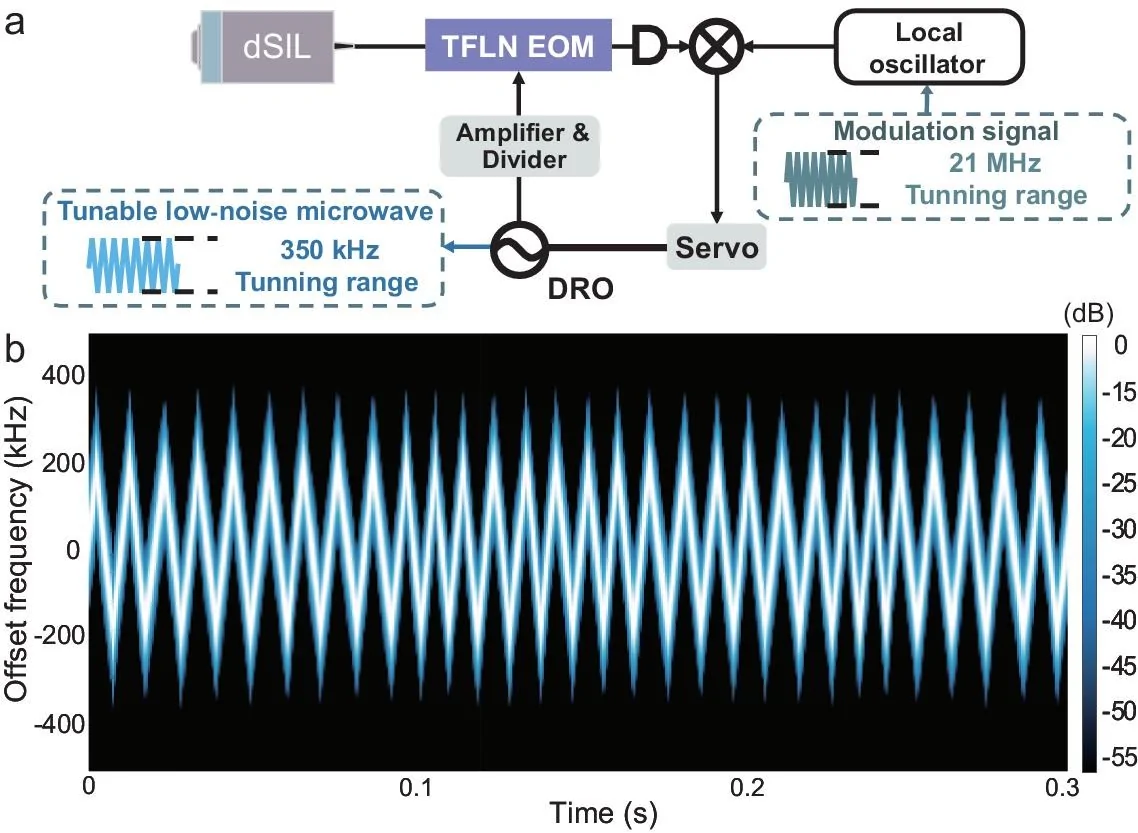
Microwave frequency agility and tuning performance. (a) Schematic of the frequency tuning test setup for locked 10.4 GHz DRO. A linear frequency modulation signal is applied to the local oscillator, enabling tuning of the output microwave frequency. (b) Measured offset frequency of the output microwave, characterized by a real-time spectrum analyzer (RBW 1 kHz).

## CONCLUSION AND OUTLOOK

While OR-PMOs have demonstrated remarkable low-noise microwave performance, efforts toward miniaturization have historically been limited by the complexity of generating a frequency-stabilized optical reference. Here, we present a compact e-OFD system based on dSIL lasers optically locked to a high-Q μFP reference cavity. The resulting frequency-stabilized optical reference is subsequently converted into dual EO combs via a monolithic TFLN cascaded modulator, enabling low-noise microwave generation with 35.5 dB frequency-division noise suppression and single-sideband phase noise of −54 dBc/Hz at 10 Hz, −141 dBc/Hz at 10 kHz, and −162 dBc/Hz at 10 MHz offset. All of this is realized within a compact, fully integrated module that effectively combines miniaturization with deployability.

In subsequent work, the Q factor of the μFP cavity can be further optimized by adjusting its end-face reflectivity, reducing roughness, or implementation of a vacuum-gap design [13–15]. Meanwhile, the performance of the EO-based division system can be further improved through two complementary strategies: first, the output microwave frequency can be easily scaled to other microwave bands by choosing a different DRO, as the repetition rate of the EO frequency comb can be continuously tuned [42]; and second, by broadening the EO comb spectrum and increasing the spectral separation between the reference lasers to enhance the frequency-division ratio [46,47]. With these advancements, better noise performance can be anticipated. On the other hand, the current modules for the optical reference and frequency divider are still limited in size by electrical packaging constraints. Further integration of the micro-optical subsystem with the TFLN chip through advanced micro-photonic coupling techniques will substantially reduce the overall footprint [15], with remaining challenges primarily associated with efficient mode-field matching, low-loss chip–to–chip coupling, and heterogeneous integration across dissimilar material platforms. Overall, our architecture not only significantly reduces the size, weight, power, and cost (SWaP-C) of the complete OFD system, but also paves the way for deployment in practical applications, including precision timing, sensing, and communication on next-generation mobile platforms [48,49].

## METHODS

We characterize the linewidths and the phase noise of the beat note using a delay-based self-heterodyne interferometer system. The dual SIL lasers are respectively injected into the self-heterodyne interferometer system. The lasers are evenly divided into two paths: one path passes through an acousto-optic frequency shifter with 40 MHz modulation frequency, while the other path passes through 1 km optical fiber to introduce an optical delay τ. After combining the two optical signals, a photodetector is used to detect the beat signal, converting the optical phase information to the RF band. We use the PNA to measure the SSB frequency noise ${S}_M( f )$ of the RF signal. Combined with the following formula (2) [44,45,50], we calculate the frequency noise ${S}_\phi ( f )$ of two lasers in self-injection locked state and free-running. The fundamental linewidths of the lasers could be calculated by π times the base noise:


(2) \begin{eqnarray*}
{S}_\phi \left( f \right) = \frac{{{S}_M\left( f \right)}}{{4si{n}^2\left( {\pi f\tau } \right)}}.
\end{eqnarray*}

Since the measured noise represents the superposition of laser noise and fiber noise, the low offset frequencies region (<5 kHz) of the test results is influenced by the residual noise from the fiber test system. To account for this, we use an additional frequency-stabilized laser to measure the low offset frequency noise of the test system (yellow curve in Fig. 3i). Nevertheless, due to the sufficient length of our delay fiber, this low offset frequency does not affect the extraction of noise information in the high-frequency region (>10 kHz) [15].

We measure the phase noise of the beat note frequency between the dSIL lasers by using the two-wavelength delayed self-heterodyne interferometer (TWDI) method [9,51–53]. When dSIL lasers simultaneously input into the self-heterodyne system, two self-heterodyne beat note signals (each with frequency shift and delay) are generated simultaneously. The key aspect of this method lies in the mixing of these two beat-note signals. The voltage noise density spectrum of the resulting DC signal contains the phase noise information of the original dual laser beat note. This phase noise can be extracted through Hilbert variation and calculated using formula (2) to obtain the phase noise of the dSIL lasers beat note.
